# Are patterns of fine-scale spatial genetic structure consistent between sites within tropical tree species?

**DOI:** 10.1371/journal.pone.0193501

**Published:** 2018-03-16

**Authors:** James R. Smith, Jaboury Ghazoul, David F. R. P. Burslem, Akira Itoh, Eyen Khoo, Soon Leong Lee, Colin R. Maycock, Satoshi Nanami, Kevin Kit Siong Ng, Chris J. Kettle

**Affiliations:** 1 Institute for Terrestrial Ecology, ETH Zurich, Zurich, Switzerland; 2 School of Biological Sciences, University of Aberdeen, Aberdeen, United Kingdom; 3 Graduate School of Science, Osaka City University, Sumiyoshi-ku, Osaka, Japan; 4 Forest Research Centre, Sabah Forestry Department, Sabah, Malaysia; 5 Forest Research Institute Malaysia, Kepong, Selangor, Malaysia; 6 Faculty of Science and Natural Resources, Universiti Malaysia Sabah, Sabah, Malaysia; Chinese Academy of Forestry, CHINA

## Abstract

Documenting the scale and intensity of fine-scale spatial genetic structure (FSGS), and the processes that shape it, is relevant to the sustainable management of genetic resources in timber tree species, particularly where logging or fragmentation might disrupt gene flow. In this study we assessed patterns of FSGS in three species of Dipterocarpaceae (*Parashorea tomentella*, *Shorea leprosula* and *Shorea parvifolia*) across four different tropical rain forests in Malaysia using nuclear microsatellite markers. Topographic heterogeneity varied across the sites. We hypothesised that forests with high topographic heterogeneity would display increased FSGS among the adult populations driven by habitat associations. This hypothesis was not supported for *S*. *leprosula* and *S*. *parvifolia* which displayed little variation in the intensity and scale of FSGS between sites despite substantial variation in topographic heterogeneity. Conversely, the intensity of FSGS for *P*. *tomentella* was greater at a more topographically heterogeneous than a homogeneous site, and a significant difference in the overall pattern of FSGS was detected between sites for this species. These results suggest that local patterns of FSGS may in some species be shaped by habitat heterogeneity in addition to limited gene flow by pollen and seed dispersal. Site factors can therefore contribute to the development of FSGS. Confirming consistency in species’ FSGS amongst sites is an important step in managing timber tree genetic diversity as it provides confidence that species specific management recommendations based on species reproductive traits can be applied across a species’ range. Forest managers should take into account the interaction between reproductive traits and site characteristics, its consequences for maintaining forest genetic resources and how this might influence natural regeneration across species if management is to be sustainable.

## Introduction

Many forest tree species possess high levels of intraspecific genetic diversity maintained by large effective population sizes, long life spans with over-lapping generations, and typically high gene flow [1,2]. Genetic diversity is not, however, evenly distributed within a species, as landscape scale genetic structure develops between subpopulations. Within subpopulations occupying contiguous local habitats, fine-scale spatial genetic structure (FSGS) may develop. FSGS is the non-random distribution of alleles through a population, and is typically observed as a negative relationship between genetic similarity and geographic distance between individuals [3].

An understanding of the processes that generate and maintain FSGS in tropical trees has direct relevance for the resilience of forest landscapes, as genetic diversity affects species’ reproductive ecology, fitness and adaptive potential [1,4]. Such knowledge is valuable in the context of the recovery of forests after selective logging, and indeed the sustainability of such logging practices. In Southeast Asia, the Dipterocarpaceae has importance for high value timber and carbon sequestration [5]. Dipterocarps typically comprise 28–53% of the total above-ground biomass [6] and account for 80% of Southeast Asia’s timber exports and 25% of global tropical hardwood consumption [7]. Consequently, these forests have been rapidly exploited over the last century, driving the region’s high rates of deforestation and forest degradation, a trend accentuated by forest conversion to agriculture [8–11]. Given post-logging low abundances of reproductive dipterocarps [12,13], and changes to their aggregation, FSGS can influence the amount of genetic diversity in subsequent fruit crops. A number of studies have identified reduced genetic diversity in logged dipterocarp populations [14,15], particularly after the second and third cutting cycles [16]. There is also a growing concern to ensure that forest genetic resources are maintained to allow resilience to logging disturbance and future climate change [17]. Increased efforts must be made to integrate this information into existing management to ensure the long-term viability of production forests in this region.

Fine-scale spatial genetic structure in the Dipterocarpaceae has been investigated by several authors [4,18–20]. Most recently, Tito de Morais et al. [18] collated data on FSGS in 19 dipterocarp species to analyze which reproductive and ecological traits underpinned the spatial scale and intensity (strength of the correlation between geographic and genetic distance) of FSGS. Species with larger flowers had limited or weaker FSGS than did smaller‐flowered species, consistent with the hypothesis of long distance pollen dispersal by larger insect pollinators [4]. Whilst seed dispersal had no significant effect on the intensity of FSGS (the strength of the correlation of relatedness with distance), the scale of FSGS (the distance to which pairs of individuals are more related than expected by chance) increased as seed dispersal potential decreased (Tito de Morais et al 2015). Their study corroborates the suggestion by Harata et al. [19] that FSGS in adult dipterocarp populations is determined primarily by seed dispersal at fine scales (<100 m) and pollen dispersal and spatial structure at wider scales (>100 m).

Whilst Tito de Morais et al. [18] assessed general patterns of FSGS relating to species traits based on their extensive multi-species, multi-site analysis, no datasets were available to compare FSGS of the same species at different sites. Cross‐site analyses are relevant as local factors such as topography, altitude, soil substrate, and disturbance dynamics, could alter the scale and intensity of FSGS [21,22]. Variation in these site-specific factors could potentially generate contrasting patterns of FSGS within the same species by influencing cluster size (potentially driven via soil associations or gap-phase regeneration) [23,24], population density and, reproductive processes [3], such as pollen dispersal distances [25,26]. The range of many common dipterocarps is extensive, with some species in the genus *Shorea*, in particular *S*. *leprosula* and *S*. *parvifolia*, possessing distributions spanning much of the Sundaland floristic region including Peninsular Thailand and Malaysia, Sumatra and Borneo [27,28]. Hence there is potential for FSGS patterns to vary widely across ranges that encompass a multitude of soil types, local climatic conditions, and forest community compositions. Analysing FSGS across sites that vary in environmental conditions within the same species allows us to investigate the effect that site conditions might have on patterns of FSGS and, the corollary of this, the extent to which FSGS can be reliably generalizable within species across sites. Such work has applied relevance as forest managers and conservationists increasingly recognize the importance of maintaining genetic diversity in forest tree populations due to its importance for adaptation to environmental change. Integrating knowledge on patterns of FSGS into management recommendations is one approach to include local genetic diversity [18,29,30].

Many Dipterocarpaceae in Southeast Asian rain forests show positive or negative habitat associations with particular soil substrates [31–37] and topography [31,35,38–41]—which covary in many lowland forest plots. These associations are driven by habitat filtering and niche differentiation often at the juvenile stage [34,36,42,43]. The impact of such associations on species composition can be profound, with plant communities at some particularly topographically heterogeneous sites stratified into three distinct floristic associations over an elevation range of < 180 m [44], and species restricted to narrow ranges of elevation and soil chemistry. Sites that are topographically homogenous (relatively flat with gentle slopes) are thus expected to have tree communities which are relatively contiguous and evenly distributed across the site. Conversely, sites that are topographically heterogeneous (highly dissected ridges and valleys) might create relatively discrete local distributions separated in space by physical barriers and unfavorable soil conditions. Such differences in relative local abundance and spatial aggregation patterns impact the behavior of pollinators and the scale of pollination events [45], and might hinder seed movement in this gyration dispersed family, thus potentially generating contrasting patterns of FSGS for the same species between different sites.

The aim of this study was therefore to assess whether patterns of FSGS are consistent within species across sites that vary in topographic heterogeneity. We hypothesize that if species traits and reproductive processes, rather than site environmental conditions, are the primary determinant of FSGS then patterns of FSGS will be identical between different sites for the same species. To test this hypothesis, patterns of FSGS for three species of dipterocarp, each from two different sites, were analyzed using identical methods allowing us to assess the consistency in scale and intensity of their FSGS patterns. Three species, *Parashorea tomentella*, *Shorea leprosula* and *Shorea parvifolia*, with differing reproductive traits, were selected, due to their high relative abundance across elevation and soil gradients.

## Methods

We thank the Sabah Biodiversity Council (SBC) for granting permits to conduct fieldwork in Sabah and the Danum Valley Management Committee (DVMC) for granting access to the Danum Valley Conservation Area (DVCA).

### Study species

*Parashorea tomentella*, *Shorea leprosula* and *Shorea parvifolia* are emergent trees reaching to 60 m in height [27] (Table 1). *Shorea parvifolia* and *S*. *leprosula* are among the most common dipterocarp species in mixed dipterocarp forests below 700 m, with distributions encompassing Peninsular Thailand and Malaysia, Sumatra and Borneo [27,46]. *Parashorea tomentella* is endemic to Borneo and abundant below 200 m on fertile clay and alluvial soils [46]. Seed dispersal in all three species is primarily local, with fruit often failing to disperse beyond the crown of the mother tree [47–50], although convective storms can distribute fruit much further [51]. There is no substantial secondary seed dispersal. All three species are predominantly outcrossed, though at low population densities *S*. *parvifolia* employs a mixed-mating system [45]. The larger-flowered *P*. *tomentella* (based on calyx width, Table 1) is predicted to possess greater pollen dispersal distances due to larger insect pollinators [4,52].

**Table 1:** The study species and selected life-history and reproductive traits.

Species	Max. height (m)^[^^46^^]^	$\sqrt{\mathrm{wing-loading}}$ ((mg·cm/s^2^)/cm^2^)^[^^53^^]^	Predicted Dispersal Distance (m)^[^^53^^]^	Calyx width (mm)^[^^52^^]^	Mating system
*Parashorea tomentella*	65	317.0	44.52	4.2	Outcrossing^[^^4^^]^
*Shorea leprosula*	60	208.7	58.42	2.2	Outcrossing^[^^54^^,^^55^^]^
*Shorea parvifolia*	65	175.0	69.61	2.3	—

### Study sites

This study compared FSGS in *P*. *tomentella*, *S*. *leprosula* and *S*. *parvifolia* from three Forest Dynamic Plots (FDP) and a Forest Reserve (FR): the Danum Valley Conservation Area 50 ha FDP (DVCA; 4°58′ N, 118°48′ E), in Sabah, Malaysian Borneo, the Lambir Hills National Park 52 ha FDP (LHNP; 4°12′ N, 114°00′ E), Sarawak, Malaysian Borneo, the Pasoh Forest Reserve 50 ha FDP (PFR; 2°59′ N, 102°19′ E), Peninsula Malaysia, and Sepilok Forest Reserve (SFR; 5°47′ – 5°52′ N, 117°55′ – 118°03′ E), Sabah, Malaysian Borneo. Climatic conditions are similar across the four plots (Table 2). Mean annual temperatures range 26.6–27.9°C and mean annual precipitation is >2000 mm except at Pasoh (1788 mm p.a.). Vegetation is broadleaf evergreen forest [56] under the ‘mixed lowland dipterocarp forest’ classification. The plots differ primarily in their topographical heterogeneity (Table 2, S1 File). PFR is the least topographically heterogeneous, with the FDP situated on an alluvial plain ranging from 70 to 95 m.a.s.l. in elevation [14,57], followed by the DVCA which ranges from 201 to 317 m.a.s.l. The LHNP plot is the most topographically and edaphically heterogeneous, comprising a number of ravines and steep escarpments ranging 100 to 244 m elevation [58], followed by SFR, which can be subdivided into low-lying alluvial areas with low mudstone hills between 15–30 m elevation, and sandstone hills reaching 100 m elevation [59].

**Table 2:** Climate data from the four study sites.

Site	Plot size (ha)	Elevation (m)^a^	MAT (°C)	MAP (mm yr^-1^)
Danum Valley Conservation Area (DVCA)	50	202–318	26.7	2282
Lambir Hills National Park (LHNP)	52	104–244	26.6	2664
Pasoh Forest Reserve (PFR)	50	70–95	27.9	1788
Sepilok Forest Reserve (SFR)	50	13–40	27.3^[^^60^^]^	3136^[^^60^^]^

This table is adapted from Anderson-Teixeira et al. [56] with the inclusion of data from a plot in the Sepilok Forest Reserve which is not part of the CTFS-ForestGEO forest dynamic plot network.

^a^Elevation data were obtained from digital elevation models (DEMs) of Danum and Sepilok, and from the original survey data from grid intersections for Lambir and Pasoh.

### Sampling and DNA extraction

Individual adult trees were sampled from the FDP at DVCA, LHNP, and PFR [14,19], which are integrated within the CTFS–ForestGEO global network of forest plots [56]. *Parashorea tomentella* sampled from SFR by Kettle et al. [4] followed a stratified sampling approach over a much larger spatial scale, and therefore a 50 ha subsection of this dataset was used (full details are provided in S2 File).

Published microsatellite genotype and coordinate datasets are available for *P*. *tomentella* from SFR [4], *S*. *parvifolia* from LHNP [19] and *S*. *leprosula* from PFR [14]. Using identical methods, we analyzed patterns of FSGS for the same species from the DVCA plot. Consistent with the comparison datasets, all individuals with a DBH > 30 cm were sampled, and coordinates recorded using a handheld GPS (Garmin GPSmap 60CSx). Cambium samples were taken using a 2 cm diameter leather punch and hammer, following the procedure of Colpaert et al. [61]. Samples were desiccated in silica gel and then stored at -4°C prior to DNA extraction. DNA was extracted from roughly 0.025g of lyphosized sample using Qiagen DNeasy™ 96-well-plate extraction system, after first milling samples to a fine powder using a Qiagen Mixer-Mill™. Details of sampling and DNA extraction from LHNP, PFR and SFR are described in the original papers [4,14,19].

### Microsatellite genotyping

The genotype of each individual was determined at six (*P*. *tomentella*) [4,62], eight (*S*. *leprosula)* [62,63] and ten (*S*. *parvifolia*) [64] nuclear microsatellite loci (S1 Table). PCR amplifications were performed on peltier thermo cyclers (Sensoquest Labcycler and Dyad Biorad). For *S*. *leprosula* and *S*. *parvifolia* each PCR reaction consisted of 1 μL of DNA template, 2 μL of 5x GoTaq reaction buffer (Promega), 0.6 μL of MgCl_2_ (25 mM), 0.2 μL dNTP mix (10 mM), 0.4 μL M13 labelled forward primer (2 mM), 1.6 μL reverse primer (2 mM), 1.6 μL of FAM labeled M-13 fluorescent dye (2 mM), 0.18 μL BSA (10 mg/mL), 0.05 μl *Taq* Polymerase (Promega) (5 U/μL) and 2.37 μL of ddH_2_0. The touchdown PCR amplification protocol for these three species consisted of an initial denaturation at 94°C for 5 minutes, followed by eight cycles of 94°C for 30s, 58°C for 45s with a reduction of 1°C each cycle, and 72°C for 30s. This was followed by 20 cycles of 94°C for 30s, 50°C for 45s, and 72°C for 30s to provide stable annealing temperatures. The protocol finished with a final eight cycles of 94°C for 30s, 53°C for 45s, and 72°C for 30s, ending with a final extension of 72°C for 10 minutes. The *P*. *tomentella* markers were labeled and hence a modified PCR reaction and amplification protocol was used. Each PCR reaction consisted of 1 μL of DNA template, 2 μL of 5x GoTaq reaction buffer (Promega), 1.2 μL of MgCl_2_ (25 mM), 0.2 μL dNTP mix (10 mM), 2.5 μL forward primer (2 mM), 2.5 μL reverse primer (2 mM), 0.18 μL BSA (10 mg/mL), 0.05 μl *Taq* Polymerase (Promega) (5 U/μL) and 0.37 μL of ddH_2_0. The touchdown PCR amplification protocol for *P*. *tomentella* markers consisted of an initial denaturation at 95°C for 2 minutes, followed by 10 cycles of 95°C for 30s, 65°C for 30s with a reduction of 1°C each cycle, and 72°C for 30s. This was followed by 30 cycles of 95°C for 30s, 55°C for 30s, and 72°C for 30s to provide stable annealing temperatures. The protocol finished with a final extension of 72°C for 30 minutes. Fragment analysis was performed on ABI 3730xl capillary sequencer (Applied Biosystems). Genotypes were scored using GeneMarker® software version 2.6.0 (SoftGenetics, PA, USA) against a LIZ 500 HD size standard. Details of microsatellite genotyping for species sampled in LHNP, PFR and SFR are described elsewhere [4,14,19].

### Analysis of genetic diversity and inbreeding

For each locus we calculated the number of alleles (*N*_*a*_), and observed (*H*_*o*_) and expected (*H*_*e*_) heterozygosity using GenAlEx 6.4 [65]. The effective number of alleles (*A*_*e*_) and the inbreeding coefficient (*F*_*IS*_) were calculated using FSTAT [66]. The effective number of alleles is sensitive to the sample size [67,68] and thus we calculated allelic richness (*A*_*r*_) using 42 randomly selected samples per species, our lowest overall sample size, to ensure comparability between populations [67,68]. Null allele frequencies were calculated using GenePop 4.2.1 [69]. All loci were highly polymorphic enabling comparison between the species (Table 1). For species with an *F*_*IS*_ > 0.15, indicating a mixed mating system, we calculated the selfing rate (*s*), *s* = (2*F*_*IS*_)/(1 + *F*_*IS*_), for each species [70].

### Characterisation of fine-scale spatial genetic structure

The following steps were conducted for all datasets. To elucidate FSGS, the spatial autocorrelation between paired samples at multiple distance classes was calculated using the relatedness coefficient (*r*) and kinship coefficient (*F*) [71] with GenAlEx [65] and SPAGeDi respectively [72]. Eleven distance classes were used. We defined four classes of 25m in the first 100m; four classes of 50m between 100 and 300m; two classes of 200m between 300 and 700m; and finally one class of 300m between 700 and 1000m. To compare the relative intensity of FSGS between species we calculated the *Sp* statistic, *Sp* = $-{\hat{b}}_{F}/(1-{\hat{F}}_{\left(1\right)})$, where $-{\hat{b}}_{F}$ is the regression slope of the kinship coefficient and ${\hat{F}}_{\left(1\right)}$ is the mean kinship coefficient, at the nearest distance class (here 25m) [73]. The scale of FSGS for each species was defined as the maximum distance at which the kinship coefficient (*F*) differed from zero (*DistF*). A nonparametric heterogeneity test [74] was applied using GenAlEx 6.4 [65] to test for significant differences in FSGS between species present at DVCA across distance classes. A sequential Bonferroni correction [75] was applied to the *P* values, which were considered significant if *P* < 0.01 [76].

A paired *t*-test (pairing within species from the different sites), was applied to test for statistical differences in the intensity of FSGS for species between sites, as observed via the *Sp* statistics. Nonparametric heterogeneity tests [74] were applied to test whether the slopes of the spatial decay in the relatedness coefficient (*r*) differed significantly between sites on a species by species basis.

### Site environmental heterogeneity

Given the difficulty in generating a robust measure of environmental heterogeneity that encapsulates the complexity of edaphic, climatic, floristic, and topographic factors at the plot level we chose plot topographical range as a proxy for environmental heterogeneity. We calculated the 95^th^ percentiles of species’ elevation range at each plot, using digital elevation models (DEMs) generated using LIDAR data (DVCA and SFR) (S1 Fig, S2 Fig) and topographic maps (LHNP and PFR) [57,58], to interpolate individual tree elevations, and used this species ‘realized’ elevation range as a proxy of habitat heterogeneity. Such an approach might not be applicable to other research sites, where habitats do not differentiate along an altitudinal gradient. In such cases soil maps or alternative factors encapsulating habitat variability would be preferable.

## Results

### Genetic diversity and inbreeding

The microsatellite loci used for analysis of the *Shorea* species sampled from DVCA were highly polymorphic, with number of alleles per locus ranging 7–24 in *S*. *leprosula* and 6–15 in *S*. *parvifolia*. Allelic richness (*A*_*r*_) was correspondingly high with values of 11.81 for *S*. *leprosula* and 6.86 for *S*. *parvifolia* (Table 3). *Parashorea tomentella* loci were less polymorphic, ranging from 6 to 11 alleles per locus, and allelic richness (5.48) was lower than the *Shorea* species. Gene diversity (*H*_*e*_) was highest in *S*. *leprosula* (0.79 ± 0.040), intermediate for *S*. *parvifolia* (0.632 ± 0.045) and lowest for *P*. *tomentella* (0.571 ± 0.063). All species were significantly inbred (Table 3), though the inbreeding coefficient (*F*_*IS*_) varied considerably from 0.108 (± 0.034) and 0.116 (± 0.22) in *S*. *parvifolia* and *S*. *leprosula* to 0.285 (± 0.430) in *P*. *tomentella*. *Parashorea tomentella* possessed a *F*_*IS*_ value of > 0.15 and selfing rate (*s*) of 0.44. Genetic diversity and inbreeding statistics for the three comparison populations are given in Table 3 and S1 Table.

**Table 3:** Summary statistics of genetic diversity and inbreeding coefficients for the three dipterocarp species from Danum Valley Conservation Area and the comparison sites (± indicates the standard error in parenthesis).

Species	Loci	*N*_*a*_ (± SE)	*A*_*r*_	*H*_*o*_ (± SE)	*H*_*e*_ (± SE)	*F*_*IS*_	*s*
*P*.* tomentella*:							
DVCA	6	6.67 ± 1.43	5.48	0.416 ± 0.07	0.571 ± 0.06	0.285**	0.44
SFR^a^	6	8.50 ± 1.57	6.57	0.580 ± 0.08	0.575 ± 0.10	-0.001	-0.002
*S*.* leprosula*:							
DVCA	8	14.38 ± 1.94	11.81	0.708 ± 0.05	0.792 ± 0.04	0.116**	–
PFR	7	13.57 ± 2.89	10.39	0.667 ± 0.05	0.736 ± 0.07	0.064**	–
*S*.* parvifolia*:							
DVCA	10	10.30 ± 1.04	6.86	0.561 ± 0.04	0.632 ± 0.05	0.108**	–
LHNP	9	15.00 ± 2.66	15.00	0.749 ± 0.05	0.819 ± 0.03	0.098**	–

Abbreviations: number of loci (Loci); mean number of alleles (*N*_*a*_); allelic richness (*A*_*r*_); observed heterozygosity (*H*_*o*_); expected heterozygosity (*H*_*e*_); inbreeding coefficient (*F*_*IS*_) and significance (** *P*<0.01); selfing rate (*s*) following Allard and Adams [70]. Allelic richness (*A*_*r*_) is calculated on a random sample of 42 individuals per species.

^a^ Data from the 50 ha subsample of the 160 ha plot at SFR (S2 File).

### Fine-scale spatial genetic structure at DVCA

**Fig 1:**
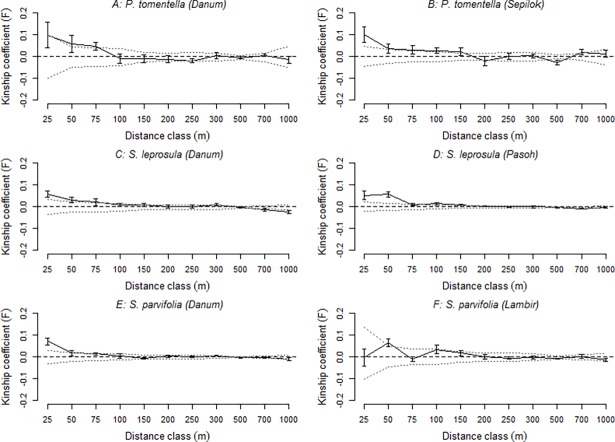
Fine-scale genetic structure of the three study species. Plots show the Kinship coefficient *F* [71] (solid line, ± standard error) plotted against distance class (m). Random spatial genetic structure at each distance class is given by the dashed line, and the 95% confidence intervals around the random spatial genetic structure by the dotted lines.

A significant correlation of *r* against geographic distance was observed in all species, confirming fine scale genetic structure in all populations sampled from the DVCA 50 ha FDP [65] (Table 4). The slopes of the regressions of *r* against the null hypothesis *r* = 0 were significant (nonparametric heterogeneity test statistic ω) for *S*. *leprosula* (ω = 90.92, *P* < 0.001), *S*. *parvifolia* (ω = 129.88, *P* < 0.001) and *P*. *tomentella* (ω = 101.46, *P* < 0.001). Significant differences in pair-wise kinship *F* [71] calculated using SPAGeDi were detected to a *DistF* of 25m in *S*. *leprosula* and *S*. *parvifolia* (*P* < 0.05) (Table 4, Fig 1). Within the smallest distance class, 0–25m, kinship values ranged from *F* = 0.058 in *S*. *leprosula* to *F* = 0.094 in *P*. *tomentella*. Despite a significant correlation of *r* against geographic distance over the full correlogram, no significant difference in *F* was observed for *P*. *tomentella* at any distance class, though a consistent trend of a reduction in *F* with distance was observed (Fig 1). The intensity of FSGS also varied between species, with the greatest intensity for *S*. *leprosula* (*Sp* = 0.015 ± 0.004) and weakest for *S*. *parvifolia* (*Sp* = 0.009 ± 0.002) (Table 4).

**Table 4:** Summary statistics (± standard error) of FSGS for *P*. *tomentella*, *S*. *leprosula*, and *S*. *parvifolia* from Danum Valley Conservation Area and the three comparison sites.

Species	Site	*N*	*F*1 (± SE)	*DistF*	bLd (± SE)	ω	*Sp* (± SE)	Elev.range
*P*.* tomentella*	DVCA	81	0.094 ± 0.06	–	-0.011 ± 0.005	43.38 **	0.012 ± 0.005	242–293
	SFR	85	0.083 ± 0.04	25	-0.023 ± 0.008	106.90 ***	0.025 ± 0.009	14–29
*S*.* leprosula*	DVCA	87	0.058 ± 0.02	25	-0.014 ± 0.004	89.73 ***	0.015 ± 0.004	235–291
	PFR	154	0.053 ± 0.02	50	-0.011 ± 0.003	90.29 ***	0.012 ± 0.003	73–85
*S*.* parvifolia*	DVCA	137	0.072 ± 0.02	25	-0.008 ± 0.002	134.87 ***	0.009 ± 0.002	242–293
	LHNP	42	-0.001 ± 0.04	–	-0.010 ± 0.004	42.03 –	0.010 ± 0.004	139–200

Abbreviations: number of samples (*N*); *F*1, mean pairwise kinship coefficient *F* among individuals at the shortest distance class (25m); *DistF*, geographic distance (m) to which *F* deviates significantly for 0; bLd, slope of the regression of pairwise kinship *F* on ln(dij), the natural logarithm of the geographic distance between pairs of individuals; ω, multi-class test criterion [74] for null hypothesis *r* = 0 (** *P*<0.01, *** *P*<0.001); *Sp*, the intensity of FSGS, following Vekemans and Hardy [73]; Elev.range, species elevation range (m) observed at the site (0.05–0.95 percentile).

Heterogeneity tests between species pairs indicated a significant difference between *S*. *parvifolia* and *P*. *tomentella* (ω = 39.73; *P* < 0.05); though significance was lost after applying the Bonferroni correction with a 1% significance threshold (*P* < 0.01) [75,76]. No difference was observed in pair-wise comparisons between *S*. *leprosula* and *P*. *tomentella* or *S*. *parvifolia*.

### Fine-scale spatial genetic structure comparisons among sites

The scale and intensity of FSGS were similar for all three species between DVCA and their comparison locations. A paired *t*-test comparing the intensity of FSGS in the DVCA populations to the comparison populations using the *Sp* statistic was non‐significant (*t* = -0.670, *P* = 0.572). Significant FSGS was observed in *S*. *leprosula* at both DVCA and PFR. The scale of FSGS was greater in PFR, with a *DistF* of 50 m compared to 25 m at DVCA. The intensity of FSGS was also slightly stronger at PFR (*Sp* value of 0.012) than at DVCA (0.015, Table 4). Nevertheless the heterogeneity test on the slope of *r* observed no significant difference between the two populations (ω = 11.38, *P* = 0.301). Similarly, levels of genetic diversity including the mean number of alleles, allelic richness, observed and expected heterozygosity and inbreeding coefficients were extremely close in value at the two sites (Table 3).

Significant though weak FSGS was observed in *S*. *parvifolia* to a *DistF* of 25 m in DVCA, but no FSGS was observed in *S*. *parvifolia* at LHNP. However, the *Sp* values measuring the intensity of FSGS were highly consistent between populations (0.009 and 0.010 respectively), and the heterogeneity test on the slope of *r* was non‐significant (ω = 9.64, *P* = 0.491). Levels of genetic diversity were higher at LHNP than DVCA, however, with greater allelic richness, mean number of alleles, and observed heterozygosity (Table 3).

The results for *Parashorea tomentella* at DVCA and SFR were less consistent. Populations from both plots exhibited significant FSGS but the intensity of FSGS was lower for the DVCA population (*Sp* value of 0.012) than the SFR population (*Sp* value of 0.025, Table 4). *Parashorea tomentella* at SFR also exhibited a significant pair-wise kinship *F* [71] calculated using SPAGeDi to a *DistF* of 25 m, while no significant pair-wise kinship *F* was observed at DVCA. A non-parametric heterogeneity test on the relatedness coefficient (*r*) across distance classes confirmed a significant difference in the pattern of FSGS between the DVCA and SFR plots for *P*. *tomentella* (ω = 40.03, *P* < 0.01). Additionally, the DVCA population was significantly inbred, with a selfing rate of *s* = 0.44 whereas the SFR population was not significantly inbred (*s* = -0.002; Table 3).

## Discussion

Our results on the scale and intensity of FSGS in *S*. *leprosula* and *S*. *parvifolia* at DVCA were highly consistent with those obtained using populations from PFR and LHNP respectively, implying little effect of topographical variation on FSGS. Conversely, the intensity of FSGS for *P*. *tomentella* was much greater at SFR than at DVCA, and a significant difference in the overall pattern of FSGS was detected between locations. Beyond assessing the consistency of species’ FSGS patterns, our aim was to assess the relative influence of site environmental heterogeneity on patterns of FSGS. Despite differences in site heterogeneity, there were no significant differences in either the intensity of FSGS on the slope of the regression between genetic relatedness and geographic distance across a pair of sites in either of the *Shorea* species.

Habitat associations in dipterocarps are thought to be maintained by niche partitioning and habitat filtering, which are likely active throughout a tree’s lifespan but are particularly intense at the juvenile stage [34,36,42,43]. Such habitat associations can lead to spatially aggregated, or clumped, distributions of adult trees on their preferred substrate, irrespective of seed dispersal potential [77,78]. Recognizing that we have data from only a limited number of sites, our data suggest that FSGS within species may be relatively invariant to site topographic heterogeneity for the two *Shorea* species, although in this study we have not considered populations that occur at the higher end of their elevational ranges, which reach 700–800 m a.s.l.

In contrast to the FSGS consistency between sites for the two *Shorea* species, results for *P*. *tomentella* showed greater intensity of FSGS at SFR than at DVCA (Table 4). Significant FSGS was also observed to a *DistF* of 25 m at SFR, but no significant *DistF* was observed at DVCA. The 50 ha plot within the SFR encompasses a much more restricted total elevation range than DVCA (37 versus 116 m), and indeed *P*. *tomentella* is restricted to a much narrower elevation range of 15 m (14–29 m) as compared to the 50 ha plot at DVCA (51 m; 242–293 m). While *P*. *tomentella* is restricted to a narrower band of low elevation areas in SFR, these areas are dissected by sandstone ridges, potentially generating a clumped spatial aggregation pattern, and driving the development of more intense FSGS in this species. Moreover, substantial soils and water regime differences are evident among these low elevation sites, as even small scale elevation differences, especially when separated by sandstone ridges, give rise to quite different edaphic conditions [60]. Evidence supporting this hypothesis is provided by Kettle et al. [4], who observed three distinct genetic clusters within this species despite a transect length of only 3 km, and mean pollen dispersal distance of 400 m. Given the increased intensity of FSGS in *P*. *tomentella* within a plot with a much more restricted elevation band, we discount the null hypothesis that species traits are the primary drivers of FSGS. This supports the idea that for some species environmental covariates across sites may influence patterns of FSGS.

Therefore, for one species, *Parashorea tomentella*, our results are consistent with the notion that habitat heterogeneity, and in particular the roughness of the terrain, can be an important factor shaping patterns of FSGS within species. This has potentially important implications for the management of genetic diversity of these commercially valuable timber tree species. For the two *Shorea* species, there was no indication that habitat heterogeneity affected FSGS. This too has relevance for forest managers as it implies that information on FSGS from one site might be generalizable across multiple sites for at least these species. Sustainable management must ensure retention of seed trees at the species level, not just the family level within lowland dipterocarp forest, if a species potential to adapt is maintained. Management recommendations such as minimum number and spatial distribution of seed trees [18] need to take account of the fact that FSGS within the same species may not always be consistent across multiple sites throughout the species’ range. Our results suggest that in one of the three species we tested, recommendations on seed tree retention and seed sampling [18] should consider the influence of site heterogeneity on patterns of FSGS. We would therefore recommend that selective logging operations are planned which take this variation among species into account. Such recommendations are timely, as current sustainability guidelines for certification (e.g. Forest Stewardship Council) place no clear requirements on selective logging or seed tree selection and retention to manage species genetic diversity.

## Supporting information

S1 Dataset Microsatellite genotype data and spatial coordinates for all trees sampled from DVCA, together with *P. tomentella* data from SFR [4], *S. leprosula* from PFR [14], and *S. parvifolia* from LHNP [18]. (XLSX)

S1 File Topographic maps of the four research plots including the coordinates of sampled individuals (S1A–S1D Fig). (DOCX)

S2 File Sampling of *Parashorea tomentella* from the Sepilok Forest Reserve (SFR). (DOCX)

S1 Fig Demographic Elevation Model (DEM) of the DVCA 50 ha FDP. Requires opening as a raster file, using the WGS84 coordinate system, UTM zone 50.
(TIF)

S2 Fig Demographic Elevation Model (DEM) of the SFR 50 ha plot. Requires opening as a raster file, using the WGS84 coordinate system, UTM zone 50.
(TIF)

S1 Table Primer details for all loci for the three dipterocarp species sampled at DVMA, together with *P. tomentella* data from SFR [4], *S. leprosula* from PFR [14], and *S. parvifolia* from LHNP [18]. (DOCX)
