# Supplementary material for: Are patterns of fine-scale spatial genetic structure consistent between sites within tropical tree species?
Source: PLoS One. 2018 Mar 16;13(3):e0193501. doi: 10.1371/journal.pone.0193501 (PMC5856272; doi:10.1371/journal.pone.0193501)

**S1 File. Topographic maps of the four research plots including the coordinates of sampled individuals (Figs S1 A-D)**

**Fig S1 A. Topographic map of the DVCA 50 ha FDP.** Rings show positions of individual sampled trees from *P. tomentella* (red), *S. leprosula* (blue) and *S. parvifolia* (yellow).

**
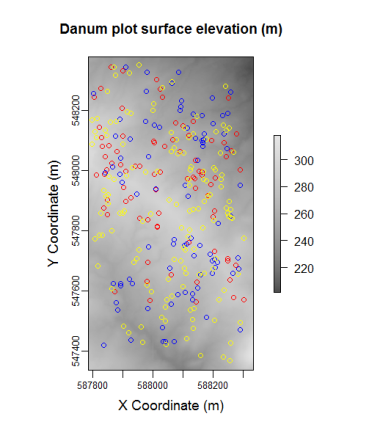
**

**Fig S1 B. Topographic map of the LHNP 52 ha FDP.** Rings show positions of *S. parvifolia* individuals sampled (yellow).

**
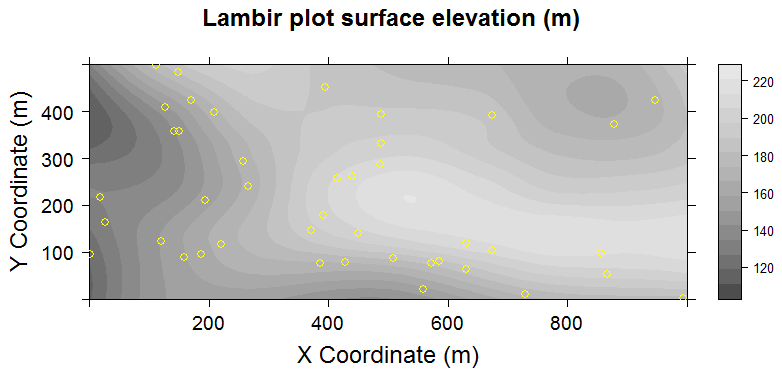
**

**Fig S1 C. Topographic map of the PFR 50 ha FDP.** Rings show positions of *S. leprosula* individuals sampled (blue).

**
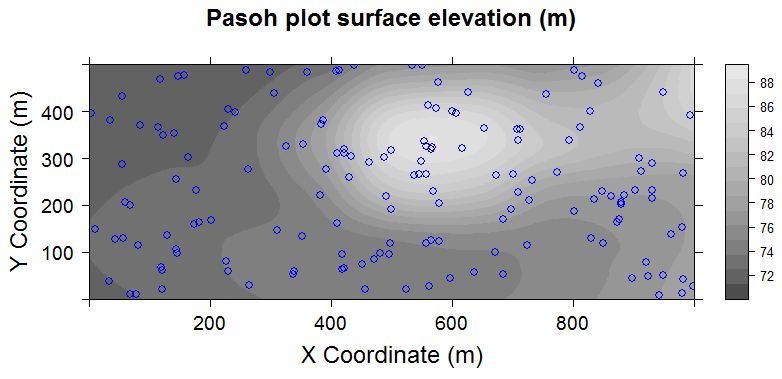
**

**Fig S1 D. Topographic map of the SFR 50 ha subsampled plot.** Rings show positions of *P. tomentella* individuals sampled (red).


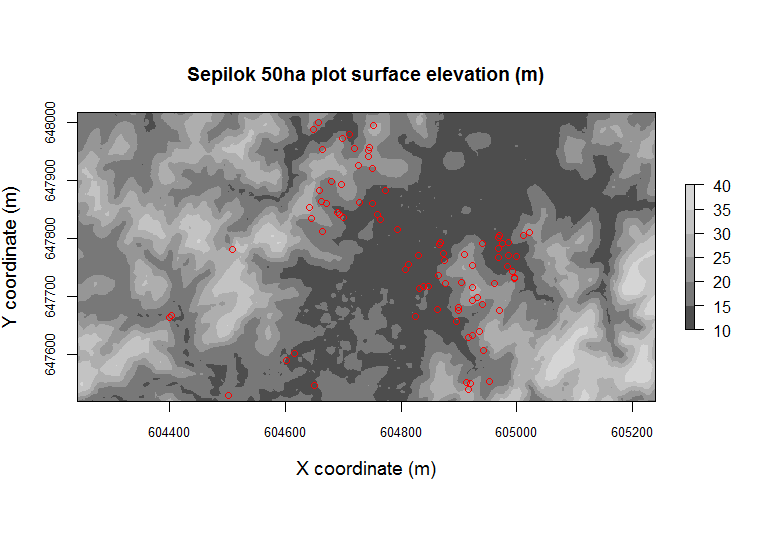

Supplement: S1 File — (DOCX) [file pone.0193501.s002.docx]
