# Supplementary material for: Are patterns of fine-scale spatial genetic structure consistent between sites within tropical tree species?
Source: PLoS One. 2018 Mar 16;13(3):e0193501. doi: 10.1371/journal.pone.0193501 (PMC5856272; doi:10.1371/journal.pone.0193501)
Supplement: S2 File — (DOCX) [file pone.0193501.s003.docx]

**S2 File. Sampling of *Parashorea tomentella* from the Sepilok Forest Reserve (SFR)**

*Parashorea tomentella* individuals sampled from SFR by Kettle et al. [4] followed a stratified sampling approach over a much larger spatial scale (to a maximum distance of 3 Km between individuals) than the CTFS 50 ha plots. Consequently this dataset contained almost three times as many individuals than are present in the DVCA 50 ha FDP. Greater genetic diversity is expected in larger populations (due to the accumulation of rare alleles) sampled over a wider spatial scale. To explicitly compare genetic diversity and FSGS between plots we therefore subsampled this larger dataset by restricting the individuals included in our analysis to those located within a 50 ha plot of equal dimensions to the CTFS plots (500 x 1000 m), delineated within the existing 160 ha plot in which all mature dipterocarps (DBH 30 cm) have been identified and coordinates recorded [61].
