# Supplementary material for: Are patterns of fine-scale spatial genetic structure consistent between sites within tropical tree species?
Source: PLoS One. 2018 Mar 16;13(3):e0193501. doi: 10.1371/journal.pone.0193501 (PMC5856272; doi:10.1371/journal.pone.0193501)
Supplement: S1 Table — (DOCX) [file pone.0193501.s006.docx]

**S1 Table. Primer details for all loci for the three dipterocarp species sampled at DVMA, together with *P. tomentella* data from SFR [4], *S. leprosula* from PFR [14], and *S. parvifolia* from LHNP [18].**

| **Species** | ***N*** | **Locus** | **Size range (bp)** | ***N_a_*** | ***A_e_*** | ***H_o_*** | ***H_e_*** | ***F_IS_*** | **Null alleles** |
| --- | --- | --- | --- | --- | --- | --- | --- | --- | --- |
| ***Parashorea tomentella*** | 81 | Dip2^a^ | 202-240 | 11 | 10.524 | 0.408 | 0.659 | 0.386** | 0.162 |
| *(Danum)* |  | Dip3^a^ | 133-139 | 3 | 2.968 | 0.416 | 0.513 | 0.196 | 0.534 |
|  |  | Dip4^a^ | 158-172 | 6 | 5.893 | 0.432 | 0.693 | 0.382** | 0.172 |
|  |  | Dip5^a^ | 178-186 | 5 | 4.988 | 0.383 | 0.416 | 0.087 | 0.099 |
|  |  | SleE02^c1^ | 162-180 | 4 | 4.000 | 0.175 | 0.385 | 0.552** | 0.187 |
|  |  | SleE16^c1^ | 193-224 | 11 | 10.795 | 0.684 | 0.759 | 0.105 | 0.066 |
|  |  | Mean |  | 6.667 | 3.347 | 0.416 | 0.571 | 0.277** | 0.203 |
|  |  | SE |  | 1.430 | 1.830 | 0.066 | 0.063 | 0.430 | 0.410 |
| ***Shorea leprosula*** | 87 | SleE02^c1^ | 153-206 | 14 | 13.829 | 0.635 | 0.755 | 0.164** | 0.092 |
| *(Danum)* |  | SleE07^c1^ | 170-187 | 7 | 6.967 | 0.674 | 0.721 | 0.070 | 0.172 |
|  |  | SleE13^c1^ | 195-254 | 20 | 20.000 | 0.827 | 0.914 | 0.102 | 0.062 |
|  |  | Sle079^c2^ | 178-246 | 24 | 23.735 | 0.938 | 0.919 | -0.013 | 0.003 |
|  |  | Sle105^c2^ | 148-174 | 10 | 9.935 | 0.500 | 0.598 | 0.169** | 0.103 |
|  |  | Sle280^c2^ | 121-171 | 15 | 14.922 | 0.766 | 0.864 | 0.120** | 0.077 |
|  |  | Sle303^c2^ | 160-193 | 14 | 13.697 | 0.733 | 0.853 | 0.147 | 0.106 |
|  |  | Sle475^c2^ | 141-169 | 11 | 10.742 | 0.595 | 0.708 | 0.166 | 0.085 |
|  |  | Mean |  | 14.38 | 14.228 | 0.708 | 0.792 | 0.111** | 0.087 |
|  |  | SE |  | 1.936 | 1.927 | 0.049 | 0.040 | 0.022 | 0.017 |
| ***Shorea parvifolia*** | 137 | Sle118^c2^ | 115-206 | 15 | 14.335 | 0.678 | 0.865 | 0.220** | 0.103 |
| *(Danum)* |  | Sle280^c2^ | 121-151 | 13 | 12.777 | 0.528 | 0.568 | 0.075 | 0.012 |
|  |  | Sle392^c2^ | 186-204 | 8 | 7.899 | 0.623 | 0.696 | 0.109 | 0.106 |
|  |  | Sle475^c2^ | 152-167 | 11 | 10.919 | 0.580 | 0.743 | 0.225** | 0.096 |
|  |  | Spar07^b^ | 132-199 | 15 | 13.536 | 0.538 | 0.584 | 0.083 | 0.043 |
|  |  | Spar11^b^ | 104-126 | 6 | 5.182 | 0.515 | 0.469 | -0.095 | 0.000 |
|  |  | Spar12^b^ | 193-229 | 9 | 9.000 | 0.820 | 0.823 | 0.007 | 0.032 |
|  |  | Spar13^b^ | 116-146 | 6 | 6.000 | 0.438 | 0.522 | 0.167** | 0.130 |
|  |  | Spar19^b^ | 232-262 | 9 | 8.441 | 0.441 | 0.463 | 0.050 | 0.071 |
|  |  | Spar20^b^ | 186-233 | 11 | 9.919 | 0.449 | 0.586 | 0.239** | 0.105 |
|  |  | Mean |  | 10.3 | 9.801 | 0.561 | 0.632 | 0.116** | 0.070 |
|  |  | SE |  | 1.044 | 0.980 | 0.038 | 0.045 | 0.034 | 0.014 |
| ***Parashorea tomentella*** | 85 | Dip02 | 207-248 | 14 | 13.578 | 0.763 | 0.861 | 0.123 | 0.066 |
| *(Sepilok)* |  | Dip03 | 132-146 | 4 | 3.977 | 0.395 | 0.374 | -0.051 | 0.087 |
|  |  | Dip04 | 152-180 | 11 | 10.170 | 0.733 | 0.723 | -0.006 | 0.023 |
|  |  | Dip05 | 172-188 | 7 | 5.657 | 0.507 | 0.419 | -0.205 | 0.000 |
|  |  | Pt05 | 109-119 | 5 | 4.678 | 0.306 | 0.307 | 0.011 | 0.035 |
|  |  | Sld19 | 178-205 | 10 | 10.000 | 0.776 | 0.764 | -0.005 | 0.028 |
|  |  | Mean |  | 8.50 | 8.010 | 0.580 | 0.575 | -0.001 | 0.040 |
|  |  | SE |  | 1.565 | 1.555 | 0.084 | 0.096 | 0.044 | 0.013 |
| ***Shorea leprosula*** | 154 | Shc01 | 132-212 | 22 | 21.701 | 0.604 | 0.911 | 0.340** | 0.014 |
| *(Pasoh)* |  | Shc02 | 135-153 | 7 | 7.000 | 0.797 | 0.603 | -0.320 | 0.000 |
|  |  | Shc03 | 121-131 | 5 | 5.000 | 0.586 | 0.578 | -0.011 | 0.050 |
|  |  | Shc04 | 80-124 | 19 | 19.000 | 0.828 | 0.906 | 0.090** | 0.047 |
|  |  | Shc07 | 129-183 | 23 | 22.940 | 0.758 | 0.911 | 0.171** | 0.093 |
|  |  | Shc09 | 170-196 | 12 | 11.880 | 0.636 | 0.803 | 0.211** | 0.106 |
|  |  | Shc17 | 65-89 | 7 | 6.945 | 0.458 | 0.441 | -0.034 | 0.015 |
|  |  | Mean |  | 13.57 | 13.495 | 0.667 | 0.736 | 0.097** | 0.069 |
|  |  | SE |  | 2.894 | 2.873 | 0.050 | 0.073 | 0.081 | 0.022 |
| ***Shorea Parvifolia*** | 42 | Dra215 | 259-318 | 28 | 28.00 | 0.881 | 0.942 | 0.076** | 0.034 |
| *(Lambir)* |  | Shc03 | 129-135 | 4 | 4.00 | 0.643 | 0.721 | 0.121 | 0.173 |
|  |  | Shc09 | 187-215 | 12 | 12.00 | 0.857 | 0.789 | -0.074 | 0.000 |
|  |  | Shc111a | 129-158 | 17 | 17.00 | 0.714 | 0.885 | 0.205 | 0.110 |
|  |  | Sle280 | 100-183 | 21 | 21.00 | 0.881 | 0.924 | 0.059 | 0.043 |
|  |  | Sle290 | 174-219 | 19 | 19.00 | 0.548 | 0.739 | 0.270** | 0.117 |
|  |  | Sle392 | 180-197 | 8 | 8.00 | 0.714 | 0.701 | -0.007 | 0.005 |
|  |  | Sle475 | 123-139 | 6 | 6.00 | 0.595 | 0.751 | 0.218 | 0.149 |
|  |  | Sle605 | 112-177 | 20 | 20.00 | 0.905 | 0.923 | 0.032 | 0.018 |
|  |  | Mean |  | 15.00 | 15.00 | 0.749 | 0.819 | 0.098** | 0.072 |
|  |  | SE |  | 2.661 | 2.661 | 0.045 | 0.033 | 0.038 | 0.022 |

Abbreviations: Number of samples (*N*); Locus name; allele size range (bp); observed number of alleles (*N_a_*); effective number of alleles (*A_e_*); observed heterozygosity (*H_o_*); expected heterozygosity (*H_e_*); inbreeding coefficient (*F_IS_*) and frequency of null alleles. *N_a_*, *H_o_*, *H_e_* were calculated with Genalex 6.5, *A_e_* and *F_IS_* with FSTAT 2.9.3.2 and frequency of null alleles with Genepop 4.2.1 (** *p*-value<0.01, **p*-value < 0.05).

^a^ Primers from Lee et al. [80] redesigned by Kettle et al. [4]

^b^ Newly developed microsatellite primers

^c^ Published primers (1) Ng et al.[63]; (2) Lee et al. [65]
